# Exploring the transition of undergraduate medical students into a clinical clerkship using organizational socialization theory

**DOI:** 10.1007/s40037-015-0241-5

**Published:** 2016-03-07

**Authors:** Anique E. Atherley, Ian R. Hambleton, Nigel Unwin, Colette George, Paula M. Lashley, Charles G. Taylor

**Affiliations:** 1 Faculty of Medical Sciences, University of the West Indies, Cave Hill Campus, Cave Hill, Barbados; 2 Chronic Disease Research Centre, University of the West Indies, Cave Hill Campus, Cave Hill, Barbados

**Keywords:** Clinical education, Transitions, Organizational socialization, Undergraduate training

## Abstract

**Background:**

Transitions in medical education are emotionally and socially dynamic; this may affect learning. Students transitioning from preclinical to clinical training may experience negative consequences. Less is understood about students’ experiences during transitions within clinical training and influential factors.

**Methods:**

The authors used organizational socialization theory to explore a transition within the clinical years. Final-year medical students experienced a nine-week internal medicine clerkship; willing students participated. Students (*n* = 101; 97 %) completed a questionnaire with open-ended questions at the beginning and end of the clerkship and participated in six consecutive focus groups, until data saturation occurred (*n* = 37). Data were thematically analyzed.

**Results:**

Socialization was challenging. Many students experienced difficulty developing relationships with team members. Students with a positive attitude experienced a smoother transition. Many students were uncertain of their roles, concerned about the workload and desired guidance to meet clerkship demands. This transition resulted in varied outcomes from enjoyment, increased confidence and student development through to disinterest.

**Conclusion:**

Transitions within clinical training are complex. Faculty should focus on adequate socialization in a new clerkship as this may facilitate a smoother transition. This may necessitate orientations, staff training, and formal student support. Further research is needed on the impact of these recommendations on learning and well-being.

## Essentials

- Students in clinical training may experience challenges while transitioning into differing environments such as clerkships; this can impact their learning.
- Orientation may be useful during the transition into a clerkship to state faculty expectations, clarify roles, foster team relationships and provide guidance.
- Faculty should focus on socialization as an important area to support students entering a clerkship; training mentors/preceptors to integrate students into teams may promote socialization.
- The transition into a clerkship can have a significant psychological impact on students; formal student support systems should be available to detect those vulnerable to learning difficulties during such a transition.
- Approaches such as longitudinal integrated clerkships may minimize fluctuations in learning produced by persistent, multiple transitions during clinical training.

## Introduction

Transitions in medical education are emotionally and socially dynamic processes [1] through which students increase expertise by acquiring new knowledge and skills [2]. Previous research on transitions in undergraduate medical education has focused on that from preclinical to clinical training. Although the transition into clinical training may be a time of significant personal and professional development [3, 4], it may also be a source of stress [5, 6] and anxiety [3–5, 7]. The latter may hinder learning [8, 9]. Some institutions have therefore implemented interventions that increase learning [10, 11] and confidence [10–13] during the transition into clinical training. Despite research on the transition into clinical training, little is understood about transitions *within* the clinical years.

Institutions with rotation-based clerkships propel students through frequent environmental changes (e.g. different clinical sites within the same institution or different institutions). These numerous transitions *within* clinical training are potentially challenging. To address these difficulties, Masters and colleagues evaluated the use of peer-to-peer handoffs to smooth transitions between dissimilar clerkship environments [2]. Other researchers have called for medical schools to re-examine the rotation-based clerkships model as frequent transitions threaten continuity of learning and socialization [14]. Theories from psychology can be useful in understanding medical education transitions [1]. Teunissen and Westerman have suggested that transitional psychology [15] or organizational socialization theory [16] may be enlightening as socialization can reduce transition-related challenges [6]. Yet, previous research into transitions during medical training has not utilized these psychology theories.

**Fig. 1:**
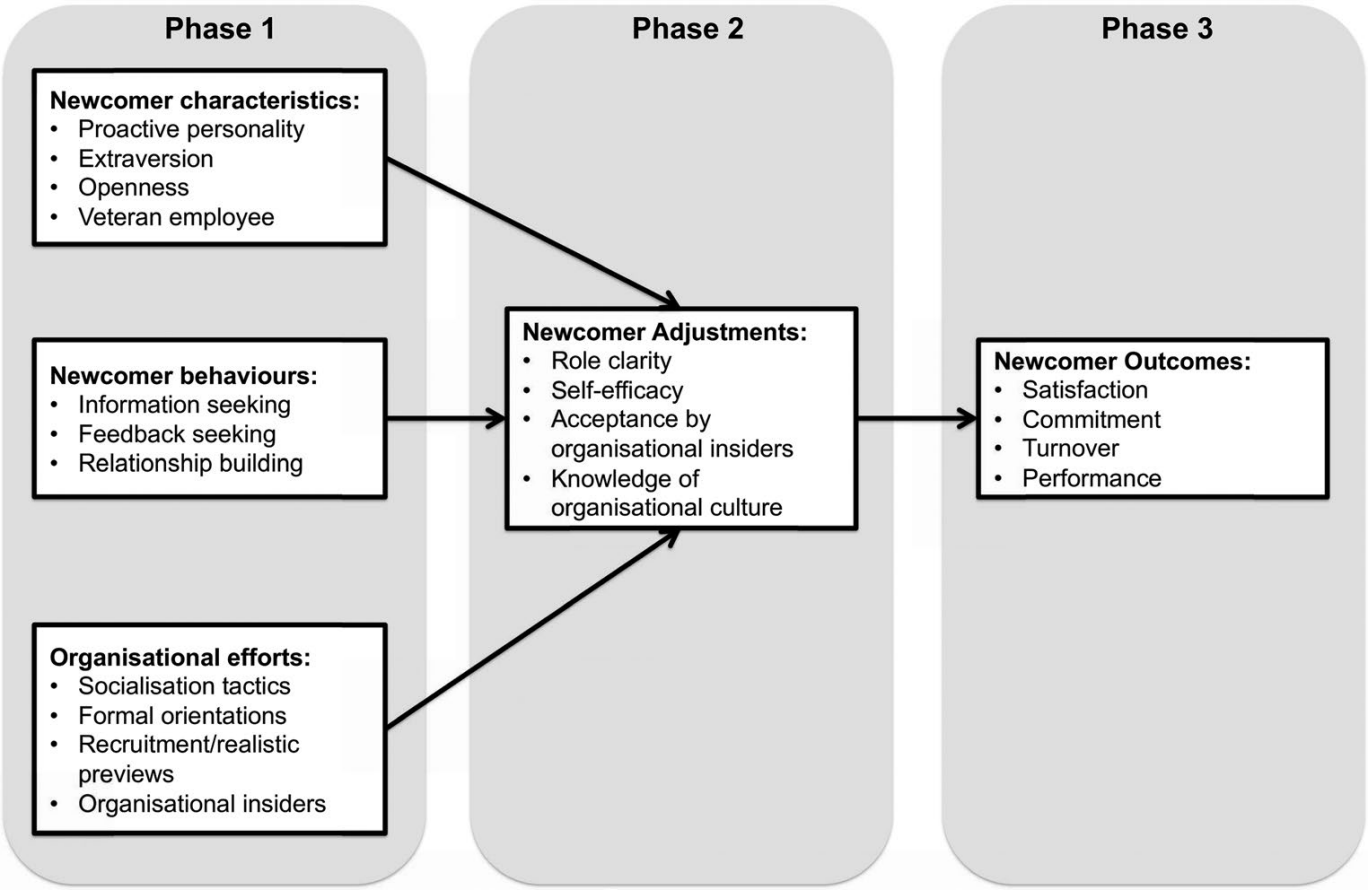
Summary diagram of the organizational socialization as described by Bauer and Erdogan [16]

We chose to use organizational socialization theory, as described by Bauer and Erdogan [16], to explore students’ experiences during the transition into a clinical clerkship. We chose this theory because medical students are deeply embedded in assigned health care social organizations during their clinical education. Nurse educators have applied organizational socialization to training in nursing [17] but little is documented on its application to medical education. Organizational socialization (Fig. 1) describes the three-phased process of a newcomer gaining knowledge, skills and behaviours that are necessary to succeed in a work environment [16]. During phase one, a newcomer’s characteristics (e.g. being proactive) increase the chance of success [16]. Seeking information and feedback, and building meaningful relationships, will also facilitate a smooth transition [16]. The organization can also influence transitions with socialization tactics (e.g. provision of mentors and support), formal orientations and allowing a preview of the work environment [16]. During phase two, factors such as role clarity, self-efficacy, acceptance by insiders and knowledge of the organizational culture determine how well the newcomer adapts [16]. Finally during phase three, Bauer and Erdogan [16] describe outcomes for the newcomer to include satisfaction, commitment, turnover and improved performance.

We underpinned our study with organizational socialization theory to explore students’ experiences during the transition into a clinical clerkship—internal medicine. We moulded conclusions about the following research questions: *(1) What were students’ experiences during the transition into a final-year internal medicine clerkship? (2) What factors did students believe affected their experiences during this transition?* Answering these questions may provide insight into how transitions *within* clinical training may affect students’ learning and well-being.

## Materials and methods

### Study design

We carried out this research under an interpretivist paradigm as we believe that there is no single truth and reality may be interpreted in different ways [18]. Organizational socialization theory [16] underpinned our analysis and discussion. We believed this theory closely approximated the reality of this transition and was likely to incorporate our findings.

### Context

The study centre was the University of the West Indies (UWI), Cave Hill Campus, which boasts a five-year medical programme (the first three years are primarily pre-clinical). Students can either enter medicine from secondary school or as graduate students; mostly the former. Our students experience three junior, introductory clerkships (internal medicine, surgery, paediatrics) during year three. Students then rotate through rotation-based clerkships primarily at the affiliated Queen Elizabeth Hospital (QEH) during the final 2 years. They navigate the final (fifth) year in peer groups of eight to fourteen through five nine-week, senior clerkships (including internal medicine). Internal medicine is only experienced at the QEH and students entering the final-year clerkship made up our sample. Students experience an orientation when transitioning into final-year internal medicine. The clerkship director incorporates a handbook to support the orientation. The orientation includes discussion of students’ expectations, students’ roles and logistics. During the final year of internal medicine, students experience ward rounds, numerous outpatient clinics and other learning opportunities including tutorials. Students spend 4–5 weeks on a clinical team before switching to another.

### Participant selection

We included students only if they rotated into the final-year senior internal medicine clerkship for the first time. We excluded elective students and those repeating the clerkship, as their previous experiences were likely to affect their current perspectives. AA, a researcher with no hierarchical relationship with the students, recruited students on the first day of each consecutive internal medicine clerkship during the study period (September 2012 to August 2014). She approached all eligible students (*n* = 104) and willing students provided written consent. A total of 37 students participated in six focus groups. Students were informed that participation would include completion of a questionnaire with two open-ended questions and possibly focus groups.

## Data collection

### Focus groups

AA conducted focus groups during the final week of each consecutive medicine clerkship using a semi-structured guideline designed by AA and CT. The interview guideline was created to allow probing for elements within phases of organizational socialization theory. Students were encouraged to reflect on their experiences during the transition into internal medicine. Discussions were stimulated with questions such as: what about entering this new environment affected you the most?’ and ‘what have you found useful in assisting the transition into the clerkship?’. We were flexible in our research design and adjusted the guideline as the study progressed based on initial analyses. Students provided further verbal consent for audio-recording of focus groups. AA recorded discussions, took written notes and transcribed recordings verbatim while removing identifiers. We aimed to have between five and eight students in each focus group to provide an environment where students would be comfortable sharing experiences [19]. We conducted focus groups until no further themes emerged.

### Open-ended questions

In addition to the focus groups, we distributed a paper, self-administered questionnaire at the beginning and end of the internal medicine clerkship to all willing, consenting students experiencing the clerkship. It contained open-ended questions that allowed triangulation with focus group data. The open-ended questions given at the beginning of the clerkship were: *(1) What concerns do you have entering this clerkship?* [Pre-1]; *(2) What do you think you will find, or have found, to be useful during the transition into this clerkship?* [Pre-2]. At the end of the clerkship, questions were: *(1) What changes have you noticed in yourself at this point in the clerkship?* [Post-1] *(2) What factors do you think influenced those changes?* [Post-2]. AA transcribed responses verbatim. We analyzed the data from the open-ended questions with focus group data as described below. No new themes were generated after students (*n* = 101; 97 %) completed questionnaires.

## Data analysis

Data were analyzed using thematic analysis. AA and CT developed a coding frame informed by organizational socialization theory and our research questions. AA and CT individually coded transcripts line by line, followed by iterative cycles of coding, code updates and thematic analysis as we discussed emerging themes. During our cycles we maintained discussion as we interpreted emerging themes through the lens of organizational socialization theory. We planned to resolve any discordance in our conclusions through discussion with CG; this was not necessary.

## Ethical consideration

Ethical approval was obtained from The University of the West Indies-Cave Hill/Barbados Ministry of Health Research Ethics Committee/Institutional Review Board.

## Results

We present our findings in relation to themes influenced by Bauer and Erdogan’s factors and phases of organizational socialization [16].

## Newcomer characteristics

Characteristics such as a positive approach and previous experience impacted the transition into internal medicine.

### Positivity

A few students considered that having a positive mind might ease their transition into internal medicine with some depending on spirituality to pull them through. *‘A strong faith in God and a belief in miracles. That is the honest truth.’[Pre-2],‘Staying relaxed, being confident and having a positive attitude…’[Pre-2]*. A few other students recognized that they were comparatively more negative and were concerned this may affect their transition. *‘…I tend to get easily frustrated and give up easily.’[Pre-1]*.

### Previous experiences

Many students felt previous experiences did not adequately prepare them for this clerkship and felt their knowledge was insufficient to successfully begin internal medicine *‘…you come into the 5th year and you’re in complete shock. Somebody dropped you in the deep end in cold water and you have to try and swim…’[Interview]*. However, some acknowledged that the knowledge and skills gained from other clerkships, particularly emergency medicine, decreased apprehension when entering internal medicine. *‘… a lot of things you do in Accident and Emergency (A&E) actually come in pretty handy on Medicine…the A&E experience before Medicine definitely helped.’[Interview]*. Very few students acknowledged how previous transitions might have assisted their approach to this one. *‘Time management skills were useful during transitions into most clerkships and will definitely assist in this one.’[Pre-2]*.

## Medical student behaviours

Students sought information on their roles and their initial performance. Many students recognized the importance of building team relationships but found it difficult. Hearsay from previous students of internal medicine negatively affected some students during the transition.

### Role and feedback seeking

Many students were concerned about their roles on teams and thought a clear explanation would have been useful. *‘…being lost as to what is expected of me.’[Pre-1], ‘…clear definition of my role as a medical student and what is expected of a final-year student.’[Pre-2]*. There were few students who looked forward to obtaining feedback during their initial period in order to ease the transition into internal medicine. *‘Discussing patients frequently with the seniors and gaining feedback would be useful…Feedback on history-taking and physical examinations.’[Pre-2],‘I think it is necessary to have feedback. But it isn’t in every team that you get feedback…’[Interview]*.

### Relationship building

Aspects surrounding building and maintaining relationships with senior doctors were prominent in this study. Many students considered that developing a relationship with team members might be useful during the transition into internal medicine with some concerned about making a good impression. *‘Getting to know team members…being a part of the team’[Pre-1]*. Many students feared that building relationships might be difficult in reality *‘Being overly ridiculed even though I am a student and have to learn.’[Pre-1]* and instead contemplated that maintaining relationships with their student peers may be fruitful.

### Medical student grapevine

Although a medical student grapevine existed where students gained information from students who had previously experienced the clerkship, most students did not find the information from this grapevine particularly useful in their transition. *‘… you have to experience it for yourself and not really go based on what everybody else says, [doing] that made it, the transition, not as difficult as one would have expected.’[Interview], ‘One thing that made it difficult is previous groups telling us about the reputations…so you already had in your mind, well this person is really hard…’ [Interview]*.

## Organizational/institutional factors

There were many areas where students felt the institution could support their transition. Some of these areas included orientation and the clerkship’s structure.

### Orientation

Some students noted that more was potentially needed for orientation with a few not even recognizing what they received as an orientation. *‘I think an orientation would be good… an orientation week would be helpful.’ [Interview]*. Some students desired other aspects to be covered during orientation such as insight into team assignments, potential topic areas and advice from previous students. *‘…an orientation would be good…in terms of how they did the topics and everything…[they could] say what [types of] patients you’re going to have’* [Interview], ‘*where the intern talks about their transition into [medicine]…what they weren’t prepared for…that would help.’ [Interview]* There were some students who, however, did note that the existing orientation would assist with their transition into internal medicine when responding to the pre-clerkship questionnaire *‘The orientation session’ [Pre-2]*.

### Clerkship timing, duration and clerkship structure

Experiencing internal medicine as the first, final-year clerkship generated anxiety among many as they were unsure of their roles and felt faculty had high expectations of them. Students who experienced internal medicine as their last final-year clerkship were anxious due to the added pressure of having no opportunity to repeat clerkship assessment prior to final examinations. *‘…the fact that it was the last one, so there is really no room for screwing up. That was kind of added pressure.’ [Interview]*. Contrarily, a few students felt that experiencing the clerkship directly before final examinations was advantageous as examination technique would be ‘fresh in their minds’.

Some students thought the nine-week duration of the clerkship was too short given their perceived learning needs. *‘Trying to learn everything you need to learn in a short space of time.’[Interview]*. Some students felt hopeless with respect to being able to achieve all that they thought they had to within the time frame. Students commented on the impact of the rotational structure as it did not allow them to ‘settle’. *‘… I think that as you begin to… then get your feet under you and settle in a little bit was when it was time for exams.’ [Interview]*. Additionally, within the internal medicine clerkship, students changed teams creating an additional transition, which seemed to negatively affect them. *‘…as soon as you get comfortable you have to switch [teams]…’[Interview]*. Even though students did not like switching teams, the thought of staying on one team brought concerns, *‘Imagine not having exposure to somebody like Dr…’ [Interview]*.

## Newcomer adjustments

Students’ adaptation to their new environment was affected by dynamics of the clinical team (organizational insiders) and guidance.

### Team relationship dynamics

Many students disclosed anxiety and concerns surrounding the team, which dominated both focus group discussions and questionnaire responses. Students’ perceptions of senior expectations generated anxiety. *‘People expecting you to know right off the bat… They expect you to know everything about Medicine from Day 1, that was difficult.’[Interview]*. Many students felt intimidated by senior doctors. When students were not intimidated, or made to feel comfortable, their anxiety was reduced. *‘Familiarity with senior team members helps reduce anxiety. Especially Dr…’s comments that we aren’t expected to be perfect and know all from the onset.’[Pre-2]*. Many students struggled to fit into their medical teams and some felt used and unimportant. *‘…a lot of the time you don’t feel like you’re part of the team, you feel like they are just using you a lot of the time …it is very frustrating.’[Interview], ‘…the academic discussion does not involve us…they talk to each other!’[Interview]*. Students who were not intimidated by their seniors felt engaged and had more positive attitudes towards the team.*‘…he makes you more involved in the team, you present to the whole team, and he doesn’t go down on you, if you don’t answer the question right he doesn’t ‘bawl’ you out’[Interview]*.

Some students had concerns that teaching varied between medical teams, relating to both the amount and content of teaching delivered on teams (especially relating to clinical examination technique). *‘We would be doing something and the first thing they would say is ‘who taught you that?’ and you don’t want to say…it puts you in a very compromising position’[Interview]*.

### Role clarification

Some students were unclear of their roles and noted that sometimes they felt like they were alone. *‘…you’re just there by yourself most of the time.’[Interview]*. However other students had better experiences and integration into the team generated a sense of worth; this had a positive psychological impact and increased engagement. *‘…you don’t feel like you’re worthless, you feel like you have a role in the team…. it’s like, oh the 5th years will do that, it’s a standard for you to present on ward rounds. They’re depending on you, so you feel some responsibility like I have to do this.’[Interview]*.

### Clerkship workload

Many students were concerned about the volume of knowledge they felt they had to acquire during the clerkship. *‘The intensity of the clerkship and the amount of diverse knowledge that is required both to take adequate histories and perform examinations as well as to reach a working diagnosis and be able to manage the patient effectively, especially if they have multiple complaints and comorbidities.’[Pre-1]*. This affected adjustment to the clerkship as students had concerns related to the diversity of topics in Medicine and many of them felt that the clerkship was the most challenging clerkship of the final year. *‘…Medicine requires the most volume of knowledge because it covers so many things … it’s the hardest rotation in the final year, if not in medical school,’[Interview]*.

### Senior guidance

Guidance was a concern for some during the clerkship. *‘…On call we didn’t really have anyone to interact with to find out what was going on…it was basically you looking at a list and clerking the patient.’[Interview]*. These students recognized that while self-directed learning was part of clinical training, guidance was critical to assist them. *‘…some people don’t really give much guidance as to if what you are self-learning is really critical at this stage…you can’t know everything.’[Interview]*.

## Newcomer outcomes

There were a variety of outcomes from transitioning into this internal medicine clerkship. These included the way students felt about internal medicine, personal and professional development and self-efficacy beliefs.

### Enjoyment versus disinterest

Some students enjoyed internal medicine despite the challenges they experienced. *‘An increase in commitment and devotion towards the practice of medicine.’[Post-2]*. Others noted that the transition into the clerkship left them jaded with disinterest and diminished work ethic. *‘Decreased morale and work ethic. This rotation is not as student-centred as it should be.’[Post-1]*.

### Personal and professional development

Some students described maturation and becoming ‘stronger’ over the course of the clerkship. *‘Became stronger as an individual.’[Post-1]*. Some also acknowledged they had begun to gain a professional identity. *‘I am developing into a more mature medical student that would allow me to function as a competent physician after graduation.’[Post-2]*.

### Increased confidence

Many students lacked confidence and felt anxious at the beginning of internal medicine *‘I would say the first four weeks were very much like you felt like a fish out of water.’[Interview]*. This anxiety related to: the team, students’ roles, feeling unprepared and workload. Many students noted that during the latter weeks of the clerkship they felt they gained knowledge, fit better into teams and felt more prepared for examinations. *‘I think we still have a long way to go but I still feel confident that we will be successful…’[Interview]*.

## Discussion

Our findings suggest that the transition into a clerkship is complex. The transition into internal medicine seemed to follow phases 1–3 as described by Bauer and Erdogan’s organizational socialization theory [16].

## Major insights

Newcomer characteristics, behaviours and organizational efforts all played a role during phase 1 of transitioning into the internal medicine clerkship. Medical students entered internal medicine with different backgrounds, personalities and experiences. Some students were proactive and had a positive outlook while others did not. Few of our students showed feedback-seeking behaviour but those who did, noted it would be useful. These findings provide further evidence to research that shows that proactive students and those who ask for feedback are more likely to experience a smoother transition [1, 16]. Many participants found socialization and building productive team relationships difficult. Medical students highlighted the importance of orientation into the clerkship and there were other organizational aspects that impacted their initial transition into internal medicine (e.g. timing of the clerkship experience and the clerkship’s duration). Some students noted that the orientation was useful in assisting their initial transition although some students desired aspects such as logistics and student expectations to be covered. A variety of factors played a role during phase 2 of the transition into internal medicine, particularly: coping with the clerkship’s workload, clarifying roles and team dynamics. Some students remained unclear of their roles despite role-seeking and most students had concerns of the perceived workload for the clerkship. Team dynamics generated anxiety in many students who felt intimidated and struggled to fit in. Despite experiences during phase 1 and 2, some students eventually enjoyed internal medicine while others remained disinterested with decreased morale. Some students eventually recognized increased confidence and development (personally and professionally).

## An adapted model

There were a few aspects from Bauer and Erdogan’s model [16] that were not apparent in our study. These included the newcomer characteristic of being a veteran employee (not applicable to this transition). This was replaced by ‘previous experiences’ being a component that impacted students’ transition during phase 1. ‘Recruitment’ was another organizational effort from Bauer and Erdogan’s model [16] that was not relevant as all students are required to participate in an internal medicine clerkship. Outcomes of the transition such as ‘commitment’ and ‘turnover’ were also not relevant to this student transition and thus were not elicited.

**Fig. 2:**
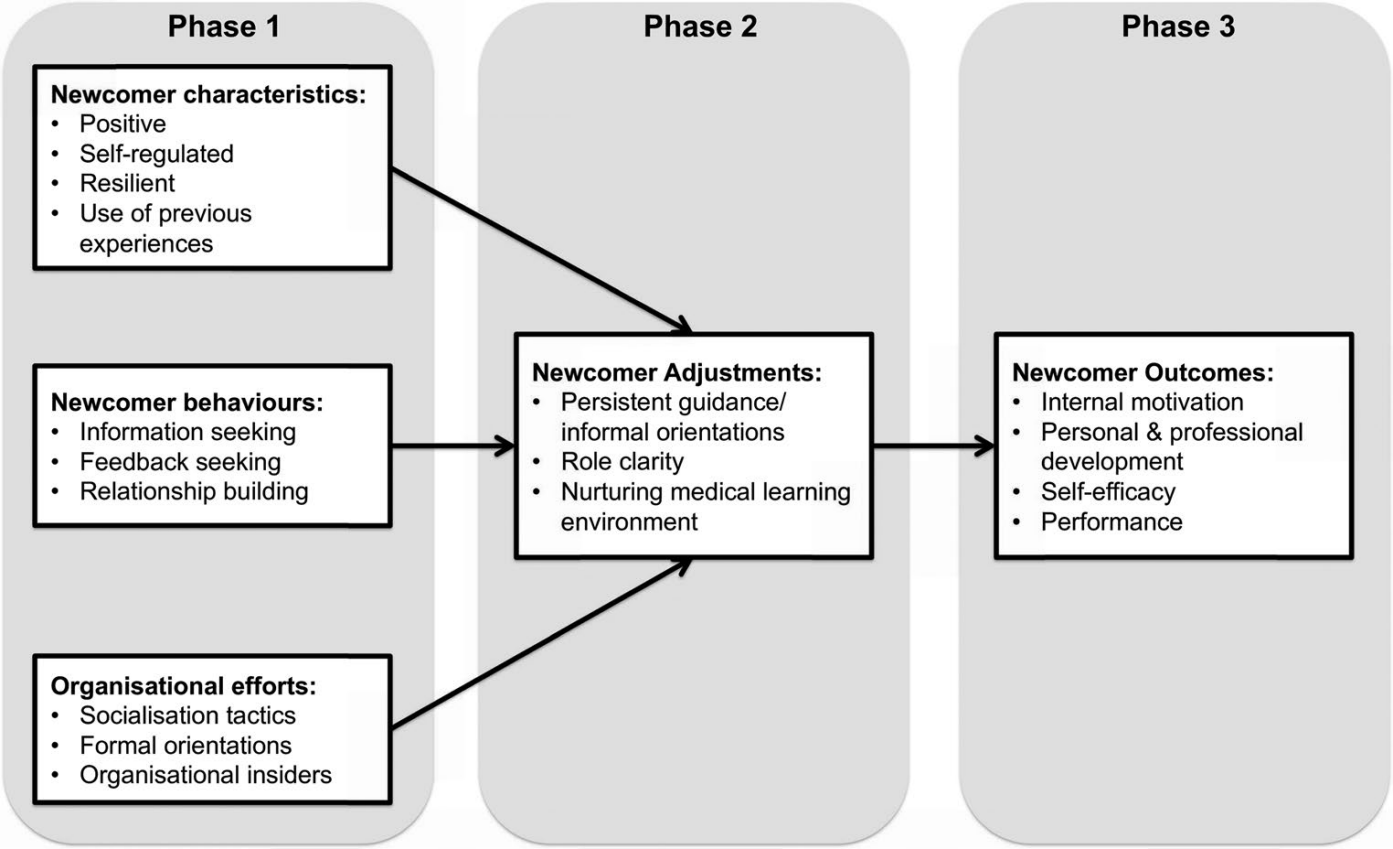
Adaptation of model of organizational socialization for a clerkship transition

We propose an adapted version (Fig. 2) to Bauer and Erdogan’s model [16] that may foster positive outcomes including: internal motivation, high self-efficacy, enhanced student development, and ultimately optimized performance in the clerkship. This adapted model may be specifically relevant to transitions in medical education. Below we discuss the implications of our findings and present recommendations incorporating our adapted model.

## Implications and recommendations

Our findings have implications for clerkship organizers and students and our recommendations follow.

### Phase 1: newcomer characteristics, newcomer behaviours and organizational efforts

As shown in our adapted model (Fig. 2), characteristics and behaviours such as being proactive and seeking feedback are likely to impact students’ initial experiences during a clerkship transition. Therefore, these should be aspects developed in our students from matriculation. Targeting skills such as self-regulated learning and resilience may prove useful in this regard. Self-regulated learning skills include goal setting, self-monitoring and self-reflection [20] and research shows that this may be useful during any transition [20]. Transitions are known to be stressful and the literature suggests that resilience—a *learned* ‘capability to thrive despite challenges’ [21]—may help students to achieve favourable outcomes despite stressful situations [22]. Developing resilience throughout the medical curriculum could reduce the potential negative impact of transitions in medical education and support students’ emotional reactions to stress. Once trained, students should make a continued effort to cultivate skills in self-regulated learning and resilience. Students should consider how previous clerkship experiences could help them in the transition between clerkships once within clinical training. Additionally, students should actively seek information about their roles and senior expectations. By increasing goal-setting, feedback-seeking behaviour may follow [23], which may further optimize students’ learning during a transition. Team members should be accommodating to facilitate students’ efforts at building relationships.

Organizational efforts to optimize clerkship transitions could include orienting students, fostering socialization and using insiders and faculty to provide student support (Fig. 2). In some cases restructuring clinical training could be beneficial though it is not always feasible. Heindenreich & Lye suggest that orienting students at the beginning of a clerkship can make learning more efficient and effective by highlighting logistics, students’ roles and supervisor expectations [24]. Similarly, Masters and colleagues found success in peer-to-peer handoffs between clerkships as an orientation exercise [2]. Although a clerkship orientation exists at our institution, it is clear that challenges remain particularly regarding logistics and student expectations. This might suggest that any intervention must be holistic and ensures all significant aspects of difficult clerkship transition are addressed. Clerkship directors should therefore consider ensuring a formal clerkship orientation to allow early dissipation of students’ anxieties, to clarify roles, to provide guidance and to foster socialization.

To further foster socialization, documented strategies of provision of mentors and student support may prove useful [16]. Training residents and preceptors to be mentors could promote socialization and reduce intimidation. Timetabled student support could also aid socialization while optimizing learning for all. Timetabled activities could include feedback, mid-clerkship briefings, study skill sessions and access to counselling [25]. These activities could potentially have a positive impact on a clerkship transition and learning. Vogan et al. note that students with poor learning or organizational skills, poor insight, mental health issues and major personal crises may be vulnerable to negative effects of transitions [25] and may require additional support of various kinds. Clerkship directors should consider screening for at-risk students to provide early interventions; this may necessitate training preceptors to recognize warning signs.

The rotation-based clerkship model may lead to educational inertia and arrested professional development’ [14]. This lack of continuity inherent in the rotation-based clerkship model is potentially detrimental to learning and both personal and professional development. Medical educators must strike a balance between clerkship duration, to allow adequate immersion, and providing exposure to an appropriate number of specialities [26]. Although one may consider longitudinal integrated clerkships—students placed in a clinical setting for extended periods [27]—many institutions may be unable to undertake such a restructuring.

### Phase 2: newcomer adjustment

Challenges during this phase included coping with the clerkship’s workload, clarifying roles and team dynamics. To optimize adjusting, clerkship directors should consider continual guidance, clarification of students’ roles and promotion of a nurturing medical learning environment (Fig. 2).

Clerkship directors should train preceptors and residents to provide continual guidance (informal orientations) [28] for all teaching experiences during the clerkship to help students cope with the workload and to clarify roles. A nurturing medical learning environment is one where students are not: fearful of making errors, mistreated, or reluctant to seek help [28]. Medical schools have a high prevalence of student mistreatment even though it may compromise well-being and learning [29]. Institutions and clerkship directors should therefore encourage a nurturing medical learning environment to support student engagement, learning affordances and guidance [30]. Having a nurturing environment may foster team building and optimize learning [4, 28]. To promote this, residents and preceptors should be aware of newcomers and should encourage learning through legitimate peripheral participation in their communities of practice [30]. Legitimate peripheral participation speaks to students’ engagement in clinical teams by initially participating in simple, low-risk tasks that are necessary and contribute to the team. Over time, students can participate in more central tasks within the team [30].

### Phase 3: newcomer outcomes

Where possible, the negative outcomes such as anxiety should be avoided. Anxiety, for example, has the potential to impact learning [15]. It may be imperative that transitions, wherever they exist, be optimized. Our recommendations may be a backbone for optimising clerkship transitions. Based on our findings and the above recommendations we propose a model to foster positive outcomes of internal motivation, high self-efficacy and enhanced student development all of which should optimize performance during the clerkship. These outcomes are measurable and could be included in clerkship evaluations.

## Limitations

This was a single-site study, which may limit transferability of these findings. We conducted focus groups retrospectively and therefore perception of earlier experiences might have been altered. To minimize this effect, we triangulated findings from focus groups with the questionnaire responses at the beginning and end of the clerkship. Specifically, questionnaire responses from the beginning of the clerkship allowed us to have data from the beginning of the clerkship to minimize the effect of perceiving earlier experiences after they had occurred. Additionally, we only explored one clerkship transition and transferability to other clerkships may be reduced. However, due to the similarity of clerkship structure, it is possible there will be similarities between experiences in other clerkships albeit with inherent environmental differences. Despite these limitations, our findings considerably resonate with the literature and the theoretical framework used. Further research is needed to evaluate the effect of our proposed model on clerkship transitions.

## Conclusion

This qualitative study revealed that the transition into a clerkship may be complex. Students experience avoidable challenges such as difficulty building team relationships, coping with the workload, and being unsure of their roles. Developing positive attributes and self-regulated learning in our students, ensuring a nurturing learning environment, providing student support and a focus on socialization may smooth the transition into a clerkship and our model may provide a background to optimize clerkship transitions in the future. Further research is needed on the impact of our recommendations on learning and well-being.
